# Assessment of Ethanolic Extraction of Chlorogenic Acid, Cynarin, and Polyphenols from Burdock (*Arctium lappa* L.) Roots Under Ultrasound

**DOI:** 10.3390/molecules29215115

**Published:** 2024-10-30

**Authors:** Yi-Chun Hsu, Chun-Yao Yang

**Affiliations:** Department of Food Science, Fu Jen Catholic University, No. 510, Zhongzheng Rd., Xinzhuang District, New Taipei City 242062, Taiwan; k95184721@gmail.com

**Keywords:** burdock roots, ultrasound-assisted extraction, chlorogenic acid, cynarin, bioactive compounds

## Abstract

The bioactive compounds in burdock (*Arctium lappa* L.) roots, including chlorogenic acid (CGA) and cynarin, are valuable for use in nutraceutical foods. The ultrasound-assisted extraction of bioactive substances from dried burdock root powder (DBR) was investigated with 95% ethanol to reduce the impact of polysaccharide inulin on the extraction of free CGA and cynarin. The ethanolic extraction of CGA and cynarin was evaluated under ultrasound (300 W) at 40 kHz (U40) and 120 kHz (U120) with shaking at 120 rpm (S120) for comparison. Using a 1/30 (g/mL-solvent) solid-to-liquid ratio at 30 °C in 120 min, amounts of CGA and cynarin with U40 were as high as 818.74 μg/g-DBR and 173.68 μg/g-DBR, respectively, being much higher than those with U120 and S120. Total phenolic content, total flavonoid content, and antioxidant activity of the extract using U40 were significantly better than using U120 and S120. For U40 and U120, CGA increased with a decreasing solid-to-liquid ratio, while cynarin showed a decrease with a decreasing solid-to-liquid ratio using U120. Moreover, no observable degradations of free CGA and cynarin in ethanol were detected. By combining ultrasound and ethanol, the extracts with high-content CGA and cynarin from burdock roots were effectively achieved for use in health foods.

## 1. Introduction

Burdock (*Arctium lappa* L.) is a medicinal plant traditionally used for the treatment of diuresis, hypoglycemia, eczema, and skin infection due to its diuretic, carminative, anti-inflammatory, antiseptic, and detoxifying properties [1,2]. Burdock roots are widely consumed as food in many countries [3,4], exhibiting hepatoprotective, antidiabetic, anticarcinogenic, antiallergic, and gastroprotective effects [5]. The bioactive compounds in burdock roots include polysaccharides, flavonoids, and polyphenols, especially the presence of caffeoylquinic acid derivatives [5,6], mainly chlorogenic acid (CGA, 3-*O*-caffeoylquinic acid) and cynarin (1,5-dicaffeoylquinic acid) with a high antioxidant activity with the benefits to human health. CGA, served as a food additive, shows the effects of prevention and treatment of metabolic syndrome, including antihypertensive, antidiabetic, and antilipidemic activities [7,8]. Cynarin exerts anti-inflammatory and anti-swelling effects in mice, and has the intervening function against glycoxidation [9,10]. Therefore, it is valuable to extract CGA and cynarin from burdock roots to be used as the source of nutraceutical foods.

However, the profile of bioactive compounds extracted from plant foods highly depends on the extraction method, the properties of the solvent, and the affinity of the components in the interface [11]. When using water as the solvent, the interaction of caffeoylquinic acids with polysaccharides during the extraction could occur and reduce the stability of free phenolic acids in the solvent by binding to polysaccharides as a complex [12,13]. In our recent publication [14], we have presented that the aqueous extraction of CGA and cynarin from burdock roots was substantially affected by the simultaneous extraction of the abundant polysaccharide inulin, which can be used as a dietary fiber and as a nutrient source for lactic acid bacteria, resulting in the amounts of free CGA and cynarin in water first increased then decreased in different extents [14,15]. The stability of free CGA and cynarin in water was reduced due to the presence of the abundant inulin, and various degrees of reduction were also related to the intensity of the ultrasound employed [14].

It is known that inulin has a fairly aqueous solubility [16]. To minimize the interaction between caffeoylquinic acids and inulin in the extraction of CGA and cynarin from burdock roots, a solvent with a polarity less than water and low solubility for inulin could be useful to enhance the stability of free CGA and cynarin. Ethanol has been investigated to be used in the precipitation of inulin and oligoglucose [17]. Although, in general, acetonitrile and acetone are more effective than ethanol to precipitate inulin, for safety reasons, ethanol is recognized to be the best choice of solvent in food applications [18]. Hence, for the purpose of extracting higher contents of CGA and cynarin without simultaneous extraction of inulin, it could be favorable to use ethanol as the solvent to avoid the interaction with inulin. 

In addition, the affinity of the compounds in the interface also plays an important role during the extraction. The extraction efficiency could be improved by promoting the transport of constituents through the network structure of the solid substrate into the solvent. This can be achieved by applying ultrasound. Ultrasound can induce cavitation effects via the generation of microstreaming to facilitate mass transfer of the constituents and is effectively applied in the extraction of bioactive substances and polysaccharides from plant-based sources [19,20,21]. However, the level of impact induced via ultrasonic irradiation on the extraction of phenolic compounds could be dependent on the frequencies and solvents used. Therefore, the aim of this study was to investigate the effects of extraction via ultrasound at different frequencies (40 kHz and 120 kHz) with 95% ethanol as the solvent on the selective ethanolic extraction of CGA and cynarin from burdock roots of the Yanagawarisou variety, without the simultaneous extraction of the abundant polysaccharide inulin into the bulk solvent. The shaking extraction mode was explored for comparison. The total phenolic content (TPC), total flavonoid content (TFC), and the ferric-reducing antioxidant power (FRAP) of the extract for different process parameters (solid-to-liquid ratio and extraction time) were explored, and the stabilities of the free CGA and cynarin during the progress of ethanolic extraction were evaluated.

## 2. Results and Discussion

### 2.1. Effect of Different Extraction Modes

The effect of different extraction modes on the ethanolic extraction of bioactive compounds from dried burdock root powder (DBR) of the Yanagawarisou variety was explored by using ultrasound at 40 kHz/300 W (U40), 120 kHz/300 W (U120), and shaking at 120 rpm (S120) with a 1/30 (g/mL-solvent) solid-to-liquid ratio, a temperature of 30 °C, and 120 min of extraction time. The results of extract yield, TPC (using gallic acid equivalent (GAE)), TFC (using rutin equivalent (RE)), and FRAP (using trolox equivalent (TE)) are shown in Table 1. It was found that the extract yield using U40 was significantly higher than that using U120 and S120, but the difference in extract yield was insignificant between U120 and S120. The TPC and TFC in the dried burdock root powder were both significantly different between the three extraction modes in the order of U40 (4.682 mg-GAE/g-DBR) > U120 (3.949 mg-GAE/g-DBR) > S120 (2.670 mg-GAE/g-DBR) for TPC and U40 (8.798 mg-RE/g-DBR) > U120 (7.547 mg-RE/g-DBR) > S120 (4.912 mg-RE/g-DBR) for TFC. In addition, the three extraction modes also presented significantly different FRAP results in the order of U40 (5.296 mg-TE/g-DBR) > U120 (3.483 mg-TE/g-DBR) > S120 (3.147 mg-TE/g-DBR), which was in accordance with the tendencies of TPC and TFC. 

In this study, by using 95% ethanol as the solvent, ultrasound exhibited a higher impact on enhancing the mass transfer of polyphenols and flavonoid compounds via the induction of microstreaming in the liquid medium, compared to the shearing effect of the shaking operation. A lower frequency (40 kHz) would provide a more intensive micro-mixing than a higher one (120 kHz). However, such a tendency in the TPC using 95% ethanol as the solvent for the extraction modes of 40 kHz, 120 kHz, and 120 rpm was somewhat different from that of using water as the solvent. As presented in our previous publication of Chen et al. (2022) [14], by using water as the solvent with the co-extraction of polysaccharide inulin, the values of TPC in the dried burdock root powder for 120 rpm and 40 kHz were only slightly higher than that for 120 kHz, with an insignificant difference [14]. This demonstrated that the severity level of the ultrasonic effect on the extraction efficiency of bioactive compounds was also dependent on the solvent properties and the interaction between the extracted constituents.

**Figure 1:**
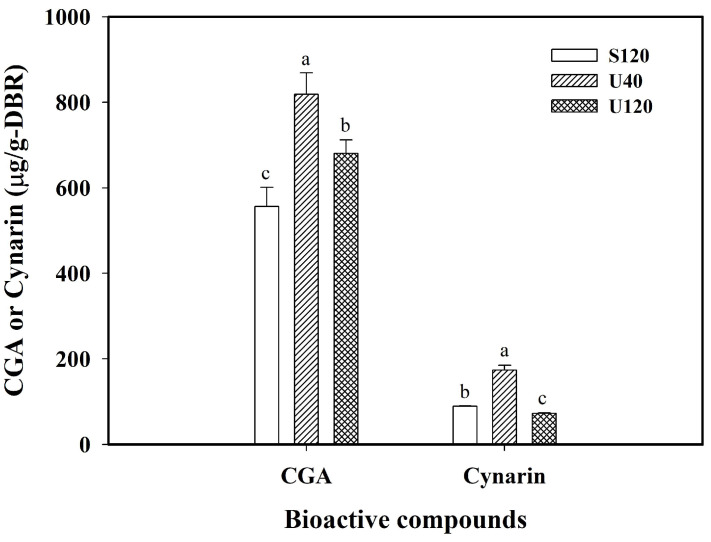
Effect of different extraction modes on the contents of chlorogenic acid (CGA) and cynarin from dried burdock root powder (DBR). Conditions: 1/30 (g/mL-solvent) solid-to-liquid ratio, 120 min of extraction time, 30 °C, using 95% ethanol as the solvent. S120: shaking at 120 rpm, U40: 40 kHz/300 W of ultrasound, and U120: 120 kHz/300 W of ultrasound. The data are expressed as mean ± standard deviations from triplicate experiments (*n* = 3). Values with different superscript letters at the same compound are significantly different (*p* < 0.05) according to Duncan’s test.

Figure 1 displays the effect of the three extraction modes (S120, U40, and U120) on the contents of free CGA and cynarin extracted from the dried burdock root powder when using 95% ethanol as the solvent to diminish the impact of polysaccharide inulin. Obviously, rather high amounts of CGA were achieved for S120, U40, and U120. The order for CGA was U40 (818.74 µg/g-DBR) > U120 (680.90 µg/g-DBR) > S120 (556.52 µg/g-DBR) with significant differences, showing that the order of CGA for U40, U120, and S120 had the same trend as that of TPC, TFC, or FRAP.

However, the intensive level of the ultrasonic effect on the extraction efficiency might vary for specific compounds under different solvent environments. By using 95% ethanol, the order for cynarin was U40 (173.68 µg/g-DBR) > S120 (89.08 µg/g-DBR) > U120 (72.63 µg/g-DBR) with significant differences. This displayed that U40 could induce a more severe cavitation effect and microstreaming to facilitate the mass transport of cynarin into the ethanol medium than U120 and S120, showing the best extraction efficiency for cynarin. Kumar et al. (2021) indicated that the ultrasonic frequency used in the extraction of bioactive compounds from plant foods and by-products was commonly between 20 and 120 kHz, and a low frequency, high intensity ultrasound generated strong shear and mechanical forces for the extraction process [22]. Moreover, unlike using water as the solvent for simultaneous extraction of phenolic acids and inulin [14], the present system used an ethanol solvent to eliminate the interaction of inulin, leading to S120 exhibiting a higher extraction efficiency for cynarin than U120. This observation for U120 might be related to the formation of free radicals in the solvent when using ultrasound. Bui et al. (2020) indicated that a low ultrasonic frequency limited the free radical formation, while a high ultrasonic frequency induced a greater level of free radical formation [23]. Moreover, the effect of cavitation-induced free radicals probably becomes more pronounced under extreme conditions or when the power and frequency are too high [24,25]. Therefore, the results demonstrated that using 95% ethanol as the solvent without the co-extraction of the polysaccharide inulin, the selective extraction of CGA and cynarin from burdock roots could be achieved by employing different ultrasonic frequencies and shaking operation. 

### 2.2. Effect of Solid-to-Liquid Ratio

**Figure 2:**
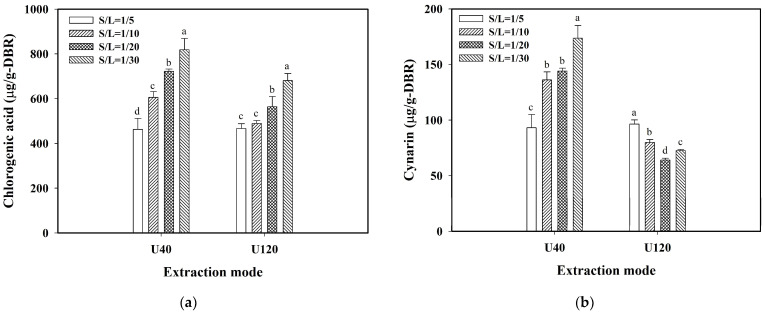
Effect of solid-to-liquid ratio (S/L) (g/mL-solvent) on the extraction of chlorogenic acid and cynarin from dried burdock root powder (DBR) by using U40 (40 kHz/300 W) and U120 (120 kHz/300 W): (**a**) chlorogenic acid content in DBR; (**b**) cynarin content in DBR. Conditions: 30 °C, 120 min of extraction time, and using 95% ethanol as the solvent. The data are expressed as mean ± standard deviation with triplicate experiment (*n* = 3). The values with different superscript letters at the same group are significantly different (*p* < 0.05) according to Duncan’s multiple range test.

The effect of the solid-to-liquid (S/L) ratio on the ethanolic extraction of bioactive compounds was explored at the conditions of 30 °C and 120 min of extraction time for the extraction modes of U40 and U120. The results of extract yield, TPC, TFC, and FRAP are shown in Table 2, and the results of CGA and cynarin are shown in Figure 2a and Figure 2b, respectively.

As displayed in Table 2, the extract yields for both U40 and U120 were significantly increased with the increase in the amount of solvent used. In addition, the values of extract yield, TPC, TFC, and FRAP when using U40 were all higher than those when using U120 for each S/L ratio. Obviously, when using 95% ethanol as the solvent, a strong effect of solvent volume on the extraction of bioactive compounds was presented. The cavitation effect at a lower S/L ratio (with more solvent) was more severe than that at a higher S/L ratio (with less solvent), leading to the increase in contact area between the solvent and the solid material, enhancing the fragmentation and erosion [22]. This demonstrated that, using 95% ethanol, the lower frequency (40 kHz) would provide a more intensive cavitation effect than the higher frequency (120 kHz) for the S/L ratio in the range of 1/5 to 1/30 g/mL-solvent. 

Figure 2a shows the content of CGA for different S/L ratios using the extraction modes of U40 and U120. It revealed that the content of CGA using U40 increased significantly with a decreasing S/L ratio, and the highest value for CGA was 818.74 µg/g-DBR at a 1/30 (g/mL-solvent) S/L ratio. The same trend was observed for CGA using U120 with a value of 680.90 µg/g-DBR at a 1/30 (g/mL-solvent) S/L ratio. The CGA values using U40 were all greater than those using U120 at the same S/L ratio. This displayed that for the extraction of free CGA into ethanol solvent, U40 could generate more intensive cavitation bubbles and micro-streaming than U120 for facilitating the transport of CGA.

For the extraction of cynarin, as shown in Figure 2b, U40 still presented the same trend as that for the extraction of CGA, increasing with a decreasing S/L ratio, and the highest value was 173.68 µg/g-DBR at a 1/30 (g/mL-solvent) S/L ratio. While the trend of increasing with decreasing S/L ratio (with more solvent) for U40 was reversed by using U120, and the highest value of cynarin using U120 was 96.375 µg/g-DBR at a 1/5 (g/mL-solvent) S/L ratio. The cause for this phenomenon was speculated as being that, for cynarin, the severity of the cavitation effect induced by U120 was not only dependent on the solvent volume but also related to the affinity of cynarin in the ethanol environment and the domination of the cavitation threshold or mechanical effect threshold in the system [26]. From the results, it was revealed that the combined effect of solvent type, S/L ratio, and different ultrasonic frequencies would be useful in the selective extraction of phenolic acids.

### 2.3. Effect of Extraction Time

**Figure 3:**
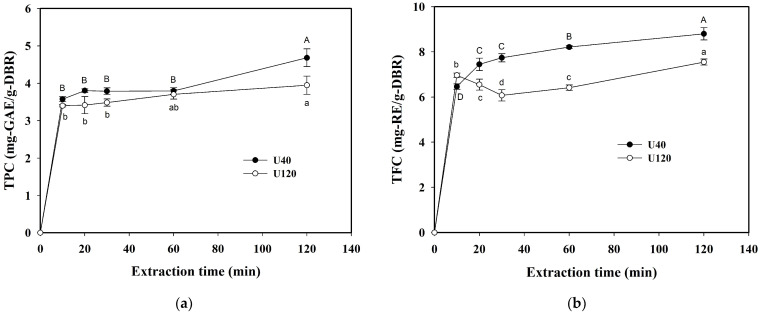
Effect of ultrasonic frequency on the variations in the total phenolic content (TPC) and total flavonoid content (TFC) against the extraction time for the extraction modes of U40 (40 kHz/300 W) and U120 (120 kHz/300 W): (**a**) TPC in DBR; (**b**) TFC in DBR. Conditions: 30 °C, 1/30 (g/mL-solvent) solid-to-liquid ratio, and using 95% ethanol as the solvent. The data are expressed as mean ± standard deviation with triplicate experiment (*n* = 3). The values with different uppercase letters for U40 and different lowercase letters for U120 are significantly different (*p* < 0.05) according to Duncan’s multiple range test.

The effect of extraction time on the extraction efficiency of phenolic compounds by using ultrasound usually relates to the stability of the extracted constituents. In this study, 95% ethanol was used as the solvent to extract the phenolic compounds without the co-extraction of polysaccharide inulin in the ethanol solvent, so as to reduce the possible degradation of the extracted compounds. Table 3 shows the results of extract yield and FRAP for the extraction time in the range of 10 to 120 min at the conditions of 30 °C and a 1/30 S/L ratio (g/mL-solvent), and Figure 3a and 3b display the variations in TPC and TFC against the extraction time using U40 and U120, respectively.

Both the extract yield and FRAP for U40 were slightly increased with the increase in extraction time, and the values were in the range of 20.93–23.67% for extract yields and 3.817–5.296 mg-TE/g-DBR for FRAP (Table 3), while for U120, the extract yield and FRAP value in 120 min were the smallest, compared to other extraction times, and the values were 20.84–23.13% for extract yields and 3.483–4.021 mg-TE/g-DBR for FRAP (Table 3). For the extraction time over 20 min, the FRAP value of the extract using U40 was larger than that using U120, showing that U40 had the benefit of extracting the constituents with more antioxidant activity from burdock roots.

As shown in Figure 3a, the values of TPC for U40 and U120 were slightly increased with the increase in extraction time. In 120 min, the TPC values for U40 (4.682 mg-GAE/g-DBR) and U120 (3.949 mg-GAE/g-DBR) were all significantly higher than those for other extraction time. Moreover, the TPC values after 20 min for U40 were all larger than those for U120. The results were consistent with FRAP values for U40 and U120 after 20 min. As displayed in Figure 3b, the TFC value for U40 was significantly increased by increasing the extraction time with the highest value of 8.798 mg-RE/g-DBR in 120 min. While for U120, the TFC value was slightly decreased in the early time of 10–30 min, but finally increased to 7.547 mg-RE/g-DBR in 120 min. Such a phenomenon in the early time was speculated to be attributed to cavitation effect and free radicals affecting some flavonoids in ethanol. 

**Figure 4:**
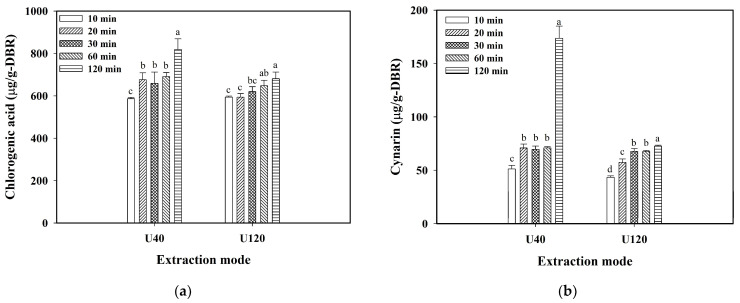
Effect of extraction time on the chlorogenic acid and cynarin extracted from dried burdock root powder (DBR) by using U40 (40 kHz/300 W) and U120 (120 kHz/300 W): (**a**) chlorogenic acid content in DBR; (**b**) cynarin content in DBR. Conditions: 30 °C, 1/30 (g/mL-solvent) solid-to-liquid ratio, and using 95% ethanol as the solvent. The data are expressed as mean ± standard deviation with triplicate experiment (*n* = 3). The values with different superscript letters at the same ultrasonic frequency are significantly different (*p* < 0.05) according to Duncan’s multiple range test.

Figure 4a,b show the variations in CGA and cynarin against extraction time in the range of 10 to 120 min, respectively. It displayed that when using either U40 or using U120, the content of CGA in 120 min was significantly higher than that in other extraction time, and the same phenomena were also observed for cynarin (Figure 4b). The results indicated that when using 95% ethanol, no observable degradations of the free CGA and cynarin were detected in the ethanol solvent, even in the presence of local hot spots induced by the cavitation effect of ultrasound, and the impact of abundant inulin of burdock roots on free CGA and cynarin extracted in ethanol was substantially diminished. The longer stabilities of free CGA and cynarin were thus obtained.

### 2.4. Effect of Extraction Mode on the Morphological Structure of Burdock Roots

**Figure 5:**
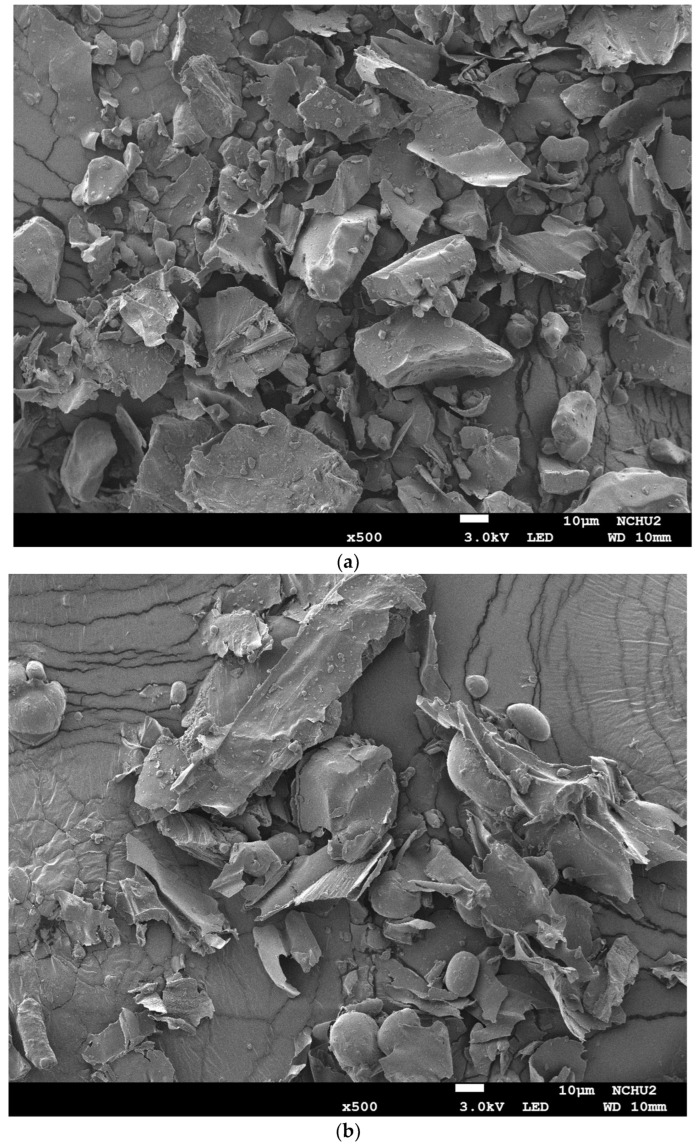

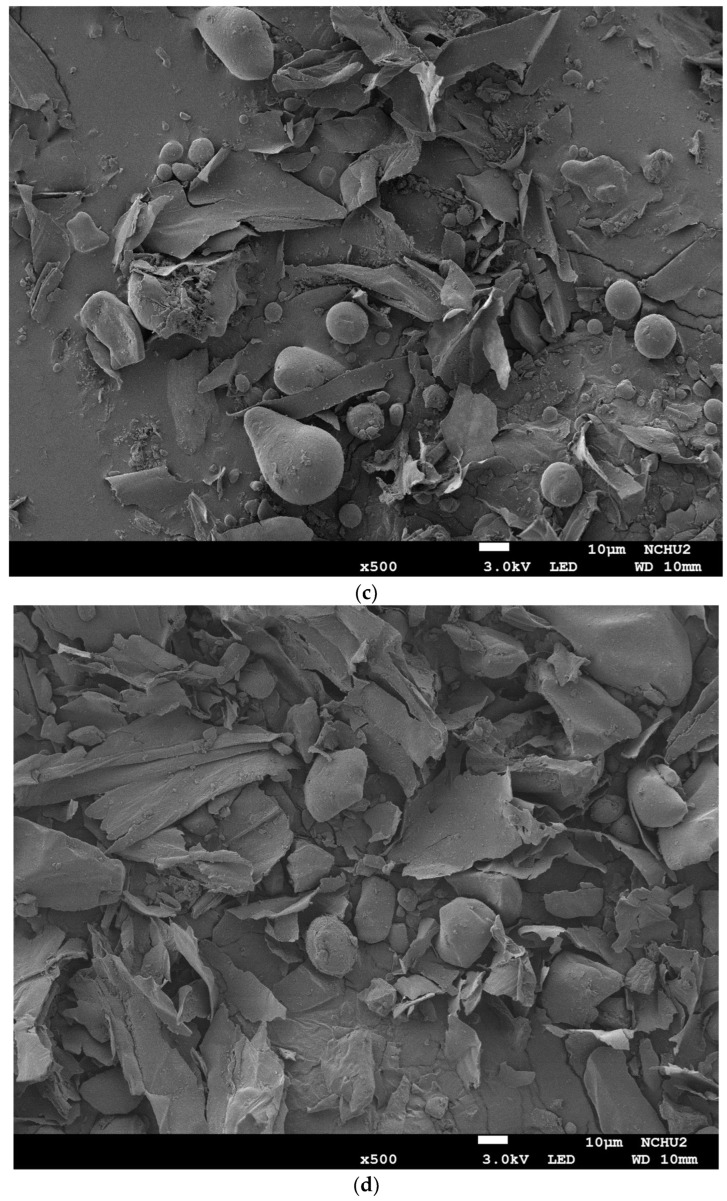
FESEM images (×500) of dried burdock root powder (DBR) and the residues of DBR via various extraction modes for the condition of 120 min of extraction and a 1/30 (g/mL-solvent) solid-to-liquid ratio at 30 °C using 95% ethanol as the solvent: (**a**) DBR; (**b**) residue of DBR by using shaking at 120 rpm (S120); (**c**) residue of DBR by using 40 kHz/300 W of ultrasound (U40); (**d**) residue of DBR by using 120 kHz/300 W of ultrasound (U120).

Figure 5 shows the images of the morphological structure of burdock roots before and after various extraction modes by using a field emission scanning electron microscope (FESEM). The microstructure of dried burdock root powder (DBR) before extraction shows a block or flake structure and some small granules attached to the surface (Figure 5a). The residues of DBR obtained via the various ethanolic extraction modes of S120, U40, and U120 are shown in Figure 5b, 5c, and 5d, respectively. From those FESEM images, the microstructures of DBR residues display spherical particles and flake structures. This phenomenon was not found in the extraction residues when using water as the solvent [14]. Burdock roots are rich in polysaccharides, and those polysaccharides (also called *Arctium lappa* L. root polysaccharide, ALP) are often precipitated with ethanol, and the structures of ALPs are mostly clumped and spherical [27]. In addition, the microstructure of DBR residue obtained by using U40 of low frequency ultrasound is more broken and fragmented than that obtained by using other extraction modes (U120 and S120). The result was caused by the cavitation effect of ultrasound, which was also reflected in the different levels of extraction efficiency for bioactive substances.

## 3. Materials and Methods

### 3.1. Materials 

The burdock (*Arctium Lappa* L.) roots of the Yanagawarisou variety purchased from Jiali District Farmers’ Association (Tainan, Taiwan) were used in this study. The fibrous root on the surface of burdock roots was first removed. Subsequently, the burdock roots were washed thoroughly, sliced, and then freeze-dried to remove water. After grinding and screening with 60-mesh sieves, the dried burdock root powder (denoted as DBR) used as the raw material for the extraction purpose was obtained and preserved away from light at 4 °C. In addition, the moisture content of dried burdock root powder (DBR) was determined as 3.98 ± 0.03% (*n* = 3) by following the method of Chen et al. (2022) [14].

The chemical reagents, 2,4,6-tris(2-pyridyl)-s-triazine (TPTZ), (±)-6-hydroxy-2,5,7,8-tetramethylchromane-2-carboxylic acid (Trolox), Folin and Ciocalteu’s phenol reagent, gallic acid, rutin, ferric chloride, and aluminum chloride were purchased from Sigma-Aldrich Co. (St. Louis, MO, USA). The standard of chlorogenic acid used in the analysis was purchased from Sigma-Aldrich Co. (St. Louis, MO, USA), and the standard of cynarin was purchased from Toronto Research Chemicals Inc. (Toronto, ON, Canada). The reagent 95% ethanol was purchased from Taiwan Sugar Corporation (Tainan City, Taiwan). Other chemicals were purchased from Sigma-Aldrich Co. (St. Louis, MO, USA), Bionavas biotechnology Co. (Toronto, ON, Canada), and Merck (Darmstadt, Germany).

### 3.2. Ethanolic Extraction Under Ultrasound and Shaking

To evaluate the efficiency of various extraction modes with 95% ethanol as the solvent, the extraction of bioactive compounds from DBR was carried out by using 40 kHz/300 W (denoted as U40) and 120 kHz/300 W (denoted as U120) of ultrasonic bath systems (LEO-3002S, LEO-3002H, LEO Ultrasonic Co., New Taipei City, Taiwan) with 0.028 W/mL of the powder density. The reciprocal shaking bath (Model B602D, Firstek, Taipei, Taiwan) at 120 rpm (denoted as S120) was also used for comparison. To start the extraction, the required quantity (2 g) of DBR was introduced into the solvent of 95% ethanol in a 150 mL flask at the setting solid-to-liquid ratio (1/5, 1/10, 1/20, or 1/30 g-DBR/mL-solvent) at the temperature of 30 °C. At the selected extraction time (10, 20, 30, 60, or 120 min), the supernatant containing the bioactive compounds was separated from the solid–liquid mixture via centrifugation (4000 rpm; 10 min). Then, the dried burdock root extract (denoted as DBE) was obtained by removing the liquid solvent from the solution with concentration under reduced pressure and subsequent freeze-drying. The yield of the extract from DBR was estimated by using Equation (1):

Extract yield (% of DBR) = (weight of DBE/weight of DBR) × 100%(1)

### 3.3. Determination of Total Phenolic Content (TPC) and Total Flavonoid Content (TFC)

For the determination of bioactive compounds in DBR, the DBE was re-dissolved in 80% methanol in the concentration of 50 mg/mL as the liquid sample for analysis. The TPC of DBE was determined via the method of Su et al. (2023) with the gallic acid equivalent (GAE), and briefly described in the following [15]. The liquid sample of DBE (250 μL) was mixed well with the same volume of Folin–Ciocalteu reagent (1 N) to react at room temperature for 5 min. Then, the above mixture was reacted with 500 μL of Na_2_CO_3_ solution for 8 min in the dark. The TPC in the DBR was determined by measuring the absorbance of the mixture using a spectrophotometer (Hitachi, Ratio Beam Spectrophotometer U-5100, Tokyo, Japan) at 730 nm and was expressed as mg-GAE/g-DBR.

The TFC in the DBR was determined according to the method of Su et al. (2023) using the rutin equivalent (denoted as RE) [15]. A quantity of 125 μL of a liquid sample of DBE, 75 μL of sodium nitrite solution (5%), and 500 μL of deionized water were mixed well to react for 5 min. Then, 37.6 μL of aluminum chloride (10%) was added into the mixture and stood still for 5 min, followed by adding 500 μL of NaOH solution (1M). The absorbance of the mixture was measured using a spectrophotometer at 510 nm to determine the TFC in the DBR. The TFC in the DBR was expressed as mg-RE/g-DBR.

The calibration curves and correlation factors were y = 0.0291x − 0.0571 (R^2^ = 0.9955) for TPC and y = 0.0011x + 0.0196 (R^2^ = 0.9998) for TFC, where x was the corresponding concentration of the standard (μg/mL), and y was the absorbance of the standard obtained after the analysis process.

### 3.4. Determination of Ferric-Reducing Antioxidant Power (FRAP)

The ferric-reducing antioxidant power (FRAP) was determined to evaluate the antioxidant activity of DBR by following the method of Wu and Yang (2023) with trolox equivalent (denoted as TE), and briefly described as follows [28]. The liquid sample of DBE was re-dissolved in 80% methanol, and the FRAP reagent was prepared and kept at 37 °C for analysis. Then, the FRAP reagent (285 μL) was mixed with the liquid sample of DBE (15 μL) and deionized water (500 μL) to react at 37 °C for 4 min. The determination of FRAP was performed via measuring the absorbance of the mixture at 593 nm, and the FRAP of the DBR sample was expressed as mg-TE/g-DBR.

### 3.5. HPLC Analysis of CGA and Cynarin

High-performance liquid chromatography (HPLC) was employed to determine CGA and cynarin in DBE, and the method of Chen et al. (2022) was followed with a slight modification [14]. The liquid sample of DBE was prepared by re-dissolving in 80% methanol, and the HPLC system was equipped with a Mightysil RP-18 GP column (5 μm, 250 mm × 4.6 mm, Kanto Chemical Co., Tokyo, Japan) and UV-VIS detector (Hitachi Chromaster 5420 UV-VIS detector, Hitachi, Ltd., Tokyo, Japan) set at a 280 nm wavelength. The mobile phase was solvent A (acetonitrile/methanol at 60:40 (*v*/*v*)) and solvent B (0.1% trifluoroacetic acid) at the flow rate of 0.8 mL/min with the gradients set as solvent A: 20 to 30% at 0 to 6 min, 30 to 40% at 6 to 16 min, 40 to 50% at 16 to 24 min, 50 to 90% at 24 to 32 min, 90 to 20% at 32 to 38 min, and 20% at 38 to 45 min.

### 3.6. Analysis of Surface Morphology

The surface morphologies of the samples of dried burdock root powder (DBR) and the residues of DBR from various extraction modes with or without ultrasound were analyzed with a field emission scanning electron microscope (FESEM) (JEOL JSM-7800F, Tokyo, Japan) of the Instrument Center of National Chung Hsing University.

### 3.7. Statistical Analysis

Each experimental result was the result of three independent samples and was expressed as mean ± standard deviation (*n* = 3). Statistical analysis used IBM SPSS Statistics 20 (IBM Corp, Armonk, NY, USA) to perform one-way ANOVA with Duncan’s multiple range test, and significant differences were set at *p* < 0.05.

## 4. Conclusions

In this study, an effective method using ethanol and ultrasound to reduce the impact of abundant inulin on the extraction efficiency of CGA and cynarin from burdock roots was developed. The effects of ultrasound at different frequencies on the extraction efficiency for TPC, TFC, CGA, and cynarin and the antioxidant activity of the extracts were analyzed and compared with shaking extraction. The solid-to-liquid ratio and extraction time were the important factors for the ethanolic extraction under ultrasound. The utilization of U40 can promote the TPC, TFC, and FRAP of the extract. The extracted CGA increased as the amount of solvent increased, but cynarin extracted via U120 decreased with the decrease in the S/L ratio. Moreover, the extracted CGA and cynarin increased with increasing extraction time without observing degradation of CGA and cynarin when using 95% ethanol as the solvent, and the impact of the abundant inulin in burdock roots on free CGA and cynarin extracted in ethanol was substantially diminished. The stabilities of free CGA and cynarin in the solvent were thus enhanced. The combination of ultrasound and ethanol could be effectively applied to extract bioactive substances from burdock roots with higher contents of CGA and cynarin to be utilized as a nutritional supplement in food application.

## Figures and Tables

**Table 1:** Effect of different extraction modes on the extract yield, total phenolic content (TPC), total flavonoid content (TFC), and ferric-reducing antioxidant power (FRAP) in the dried burdock root powder (DBR).

Extraction Mode	Extract Yield (% of DBR)	TPC (mg-GAE/g-DBR)	TFC (mg-RE/g-DBR)	FRAP (mg-TE/g-DBR)
S120	20.97 ± 0.67 ^b^	2.670 ± 0.167 ^c^	4.912 ± 0.299 ^c^	3.147 ± 0.138 ^c^
U40	23.67 ± 0.36 ^a^	4.682 ± 0.237 ^a^	8.798 ± 0.274 ^a^	5.296 ± 0.146 ^a^
U120	20.84 ± 0.24 ^b^	3.949 ± 0.242 ^b^	7.547 ± 0.134 ^b^	3.483 ± 0.108 ^b^

Conditions: 1/30 (g/mL-solvent) of solid-to-liquid ratio, 120 min of extraction time, 30 °C, using 95% ethanol as the solvent. S120: shaking at 120 rpm, U40: 40 kHz/300 W of ultrasound, and U120: 120 kHz/300 W of ultrasound. The data are expressed as mean ± standard deviations from triplicate experiments (*n* = 3). Values with different superscript letters at the same column are significantly different (*p* < 0.05) according to Duncan’s test.

**Table 2:** Effect of solid-to-liquid ratio (S/L) on the extract yield, total phenolic content (TPC), total flavonoid content (TFC), and ferric-reducing antioxidant power (FRAP) from the dried burdock root powder (DBR) by using U40 and U120.

Extraction Mode	S/L Ratio (g/mL-Solvent)	Extract Yield (% of DBR)	TPC (mg-GAE/g-DBR)	TFC (mg-RE/g-DBR)	FRAP (mg-TE/g-DBR)
U40	1/5	13.31 ± 1.95 ^d^	2.563 ± 0.178 ^c^	5.242 ± 0.533 ^c^	3.133 ± 0.276 ^c^
	1/10	15.93 ± 0.62 ^c^	3.214 ± 0.278 ^b^	6.957 ± 0.299 ^b^	3.939 ± 0.123 ^b^
	1/20	19.07 ± 0.41 ^b^	3.501 ± 0.116 ^b^	8.572 ± 0.157 ^a^	4.031 ± 0.104 ^b^
	1/30	23.67 ± 0.36 ^a^	4.682 ± 0.237 ^a^	8.798 ± 0.274 ^a^	5.296 ± 0.146 ^a^
U120	1/5	12.29 ± 1.76 ^d^	2.480 ± 0.254 ^c^	5.151 ± 0.484 ^c^	3.076 ± 0.341 ^b^
	1/10	15.26 ± 0.32 ^c^	2.462 ± 0.017 ^c^	6.029 ± 0.093 ^b^	3.082 ± 0.124 ^b^
	1/20	17.25 ± 0.42 ^b^	3.270 ± 0.130 ^b^	5.982 ± 0.286 ^b^	2.935 ± 0.142 ^b^
	1/30	20.84 ± 0.24 ^a^	3.949 ± 0.242 ^a^	7.547 ± 0.134 ^a^	3.483 ± 0.108 ^a^

Conditions: 30 °C, 120 min of extraction time, and using 95% ethanol as the solvent. U40: 40 kHz/300 W of ultrasound and U120: 120 kHz/300 W of ultrasound. The data are expressed as mean ± standard deviation with triplicate experiment (*n* = 3). Values with different superscript letters at the same row are significantly different (*p* < 0.05) according to Duncan’s multiple range test.

**Table 3:** Effect of extraction time on the extract yield and ferric-reducing antioxidant power (FRAP) from the dried burdock root powder (DBR) for U40 and U120.

Extraction Mode	Extraction Time (min)	Extract Yield (% of DBR)	FRAP (mg-TE/g-DBR)
U40	10	20.93 ± 0.24 ^c^	3.817 ± 0.022 ^c^
	20	22.74 ± 0.42 ^b^	4.564 ± 0.077 ^b^
	30	22.82 ± 0.60 ^b^	4.729 ± 0.284 ^b^
	60	23.65 ± 0.30 ^a^	4.739 ± 0.063 ^b^
	120	23.67 ± 0.36 ^a^	5.296 ± 0.146 ^a^
U120	10	21.47 ± 0.08 ^b^	3.876 ± 0.024 ^a^
	20	21.67 ± 0.85 ^b^	3.863 ± 0.103 ^a^
	30	22.69 ± 0.58 ^a^	4.014 ± 0.168 ^a^
	60	23.13 ± 0.37 ^a^	4.021 ± 0.087 ^a^
	120	20.84 ± 0.24 ^b^	3.483 ± 0.108 ^b^

Conditions: 30 °C, 1/30 solid-to-liquid ratio (g/mL-solvent), and using 95% ethanol as the solvent. The data are expressed as mean ± standard deviation with triplicate experiment (*n* = 3). U40: 40 kHz/300 W of ultrasound and U120: 120 kHz/300 W of ultrasound. The values with different superscript letters at the same column for the same extraction mode are significantly different (*p* < 0.05) according to Duncan’s multiple range test.

## Data Availability

Data are contained within the article.
